# NSCLC patients with rare *EGFR* Ex19del/G724S mutation showed good response to afatinib combined with chemotherapy treatment: A two-case report

**DOI:** 10.3389/fonc.2022.1054593

**Published:** 2022-11-24

**Authors:** Huilin Wang, Qitao Yu, Lina Shi, Qinhan Hou, Liang Dan, Chuqiao Liang, Xiaoyu Hong, Yun Zhao, Ruiling Ning

**Affiliations:** ^1^ Department of Medical Oncology of Respiratory, Guangxi Medical University Cancer Hospital, Nanning, Guangxi, China; ^2^ Medical Department, Nanjing Geneseeq Technology Inc., Nanjing, Jiangsu, China; ^3^ Department of Neurosurgery, Guangxi Medical University Cancer Hospital, Nanning, China; ^4^ Department of Oncology, Guangxi Zhuang Autonomous Region Ethnic Hospital (i.e. Ethnic Hospital Affiliated to Guangxi Medical University), Nanning, Guangxi, China

**Keywords:** EGFR G724S, EGFR Ex19del, non-small cell lung cancer, afatinib, chemotherapy

## Abstract

*EGFR* G724S mutation in exon 18 has been shown to be resistant to both first- and third-generation epidermal growth factor receptor tyrosine kinase inhibitors (EGFR-TKIs). However, we found a rare mutation of *EGFR* Ex19del/G724S in two patients with lung cancer who demonstrated a favorable response to the combination of afatinib and chemotherapy. Identified by next-generation sequencing (NGS), *EGFR* G724S was found from a primary and a secondary tumor biopsy, respectively. Treated with afatinib combined with chemotherapy, both patients responded well and achieved progression-free survival. Analysis of acquired mutations developed during treatment using afatinib revealed that the emergence of *EGFR* T790M or *ALK* fusion was the potential mechanism of afatinib resistance. Our study lends credence to treatment using afatinib combined with chemotherapy as a viable option for patients with Ex19del/G724S.

## Introduction

Targeted therapy with epidermal growth factor receptor tyrosine kinase inhibitors (EGFR-TKIs) is the standard first-line treatment for advanced *EGFR*-mutated non-small cell lung cancer (NSCLC). The most common EGFR mutations (nearly 85%–90%) in NSCLC patients are L858R and exon 19 deletions (Ex19del), which are defined as classical mutations. The remaining 10%–15% of EGFR mutations are non-classical mutations (1). *EGFR* G724S mutation in exon 18 is a rare driver mutation, only found in about 0.3%–0.8% of NSCLC patients. Seventy-five percent of G724S-positive patients harbored a concurrent mutation of exon 19 del and insertions (2). It has been reported that the presence of Ex19del reduced osimertinib-binding affinity to *EGFR in vitro (* 3, 4), whereas it retained sensitivity to afatinib, suggesting a possible therapy for patients (3). According to the public cases, five patients with Ex19del/G724S treated with afatinib monotherapy achieved an average of only four months of progression-free survival (PFS)(2). However, afatinib combined with chemotherapy for the treatment of Ex19del/G724S mutations has not been reported.

In this study, we reported two patients with *EGFR* Ex19del/G724S lung cancer who appeared in remission after being treated with afatinib and chemotherapy. Plasma or cerebrospinal fluid samples of these patients were then sequenced after treatment using a 139-lung-cancer-gene capture-based targeted panel (Geneseeq), aiming to comprehensively profile their concurrent mutation statuses and explore the potential resistance mechanisms in G724S-positive patients. PFS was defined as the start of afatinib therapy until the date of disease progression or date of death.

## Case presentation

### Case 1

**Figure 1:**
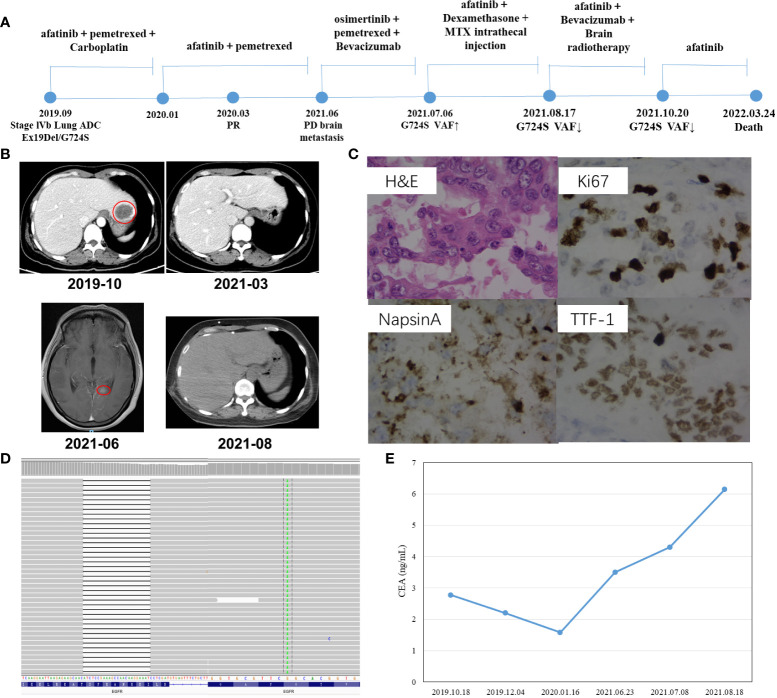
Schematic of treatment history of Case 1. **(A)** The timeline of treatment, the time point of surgery, and the NGS test are labeled above. **(B)** CT scans are shown where the red circles highlight the location of tumors. **(C)** Pathological examination of the surgical specimen. IHC testing (400×) results showed positive expression of Napsin A, TTF-1, and Ki67. **(D)** The EGFR Ex19Del/G724S mutation was visualized by IGV software. Deletions are indicated with a black dash (–). **(E)** Line chart shows changes in CEA levels in the serum tumor biomarker during the treatment.

A 47-year-old Chinese female patient without a smoking history was preliminarily diagnosed with right lung cancer with suspected multiple liver and bone metastasis. She received right upper lobe lobectomy, mediastinal lymph node dissection, pleural adhesion cauterization, phrenic nerve compression, and ninth-rib resection in September 2019 in a local hospital. However, the level of the serum tumor biomarker, carcinoembryonic antigen (CEA), was in the normal range of < 5.0 ng/ml (**Figure 1E**), and right lung adenocarcinoma (pT2N2M1c, stage IVb) was diagnosed *via* histological examination (**Figure 1C**); the result of next-generation sequencing (NGS) revealed *EGFR* Ex19del/G724S coexisting with *TP53* R158del mutation (**Figure 1D**). The patient then received afatinib treatment (30 mg qd) along with six cycles of pemetrexed and carboplatin chemotherapy (**Figure 1A**); then 14 cycles of pemetrexed were given, in consideration of the poor response to EGFR-TKIs when *EGFR* and *TP53* mutation coexist. However, adverse events of grade 2 severity (according to CTCAE 4.0) only included paronychia. Regular zoledronic acid treatment and carboplatin injection were stopped in January 2020. A partial response (PR) was seen on CT scans taken in March 2020 (**Figure 1B**), the liver tumor had disappeared, and the bone metastasis was stable, whereas a worsening brain metastasis had occurred (**Figure 1B**). In June 2021, the patient showed nuchal rigidity. Cerebrospinal fluid and plasma were then sent for NGS examination, which confirmed the existence of *EGFR* G724S mutation (AF = 1.8%) (**Table 1**) in cerebrospinal fluid, while the existence of the mutation in the plasma was negative. It has been reported that osimertinib could be a favorable option for brain metastasis, yet the symptoms were not relieved when the patient was treated with osimertinib (80 mg qd) combined with pemetrexed and bevacizumab to target the *EGFR* G724S mutation (AF = 16%). Once again, the patient received afatinib (40 mg qd) combined with MTX intrathecal injection to relieve brain symptoms and stopped osimertinib. However, the therapeutic effect was lower than expected. Ventriculoperitoneal shunt and Ommaya implantation were performed in neurosurgery in August 2021, and whole-brain radiotherapy was consequently performed in another hospital. Another cerebrospinal fluid test revealed *EGFR* G724S mutation (AF = 0.3%) in October 2021; the patient received afatinib until she died.

**Table 1:** Genetic alterations detected in cerebrospinal fluid of Case 1.

Genes	Coding change	Alternations	MAF 2021/6/10	MAF 2021/7/12	MAF 2021/8/17	MAF 2021/10/20
EGFR	c.2253_2276del	p.S752_I759del	1.30%	10.80%	1.40%	0.10%
EGFR	c.2170G>A	G724S	1.80%	16.00%	3.40%	0.30%
TP53	c.472_474del	p.R158del	0.30%	13.90%	2.20%	–
GNAS	c.728C>T	p.P243L	–	4.50%	0.60%	–

-, not detected; MAF, mutant allele frequency.

### Case 2

**Figure 2:**
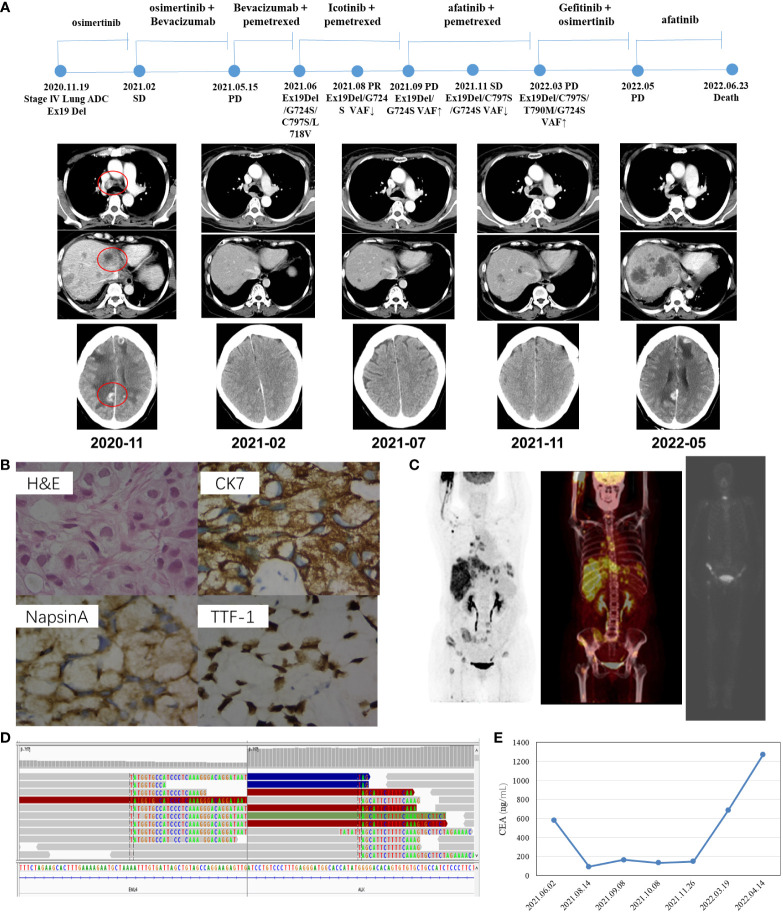
Schematic of treatment history of Case 2. **(A)** The timeline of treatment and CT scans are shown where the red circles highlight the location of tumors. The time point of NGS detected is labeled above. **(B)** Pathological examination of the surgical specimen. The H&E staining image (400×) showed a poorly differentiated adenocarcinoma histology, and IHC testing results showed a positive expression of Napsin A, TTF-1, and CK7, respectively. **(C)** Baseline positron emission tomography (PET-CT and ECT) scans with circled tumor location. **(D)** Identification of the EML4–ALK fusion. Sequencing reads of ALK and EML4 are visualized by the IGV software. **(E)** Line chart showed changes in CEA levels in the serum tumor biomarker during the treatment.

Another patient, a 61-year-old non-smoking Chinese female patient, whose mother died from lung cancer, presented to our hospital with a chief complaint of lumbago in November 2020. CT images revealed right lung adenocarcinoma with liver (**Figure 2A**), bone, and brain metastases (**Figure 2C**), with the right supraclavicular region, mediastinum, and right hilar lymph nodes spreading. Aspiration liver biopsy and IHC analysis revealed mucinous adenocarcinoma with a positive expression of Napsin A, TTF-1, and CK7 (**Figure 2B**). The PCR-based hotspot gene test of the aspiration liver biopsy revealed *EGFR* Ex19del (p.E746_S752delinsV). The patient was diagnosed with right lung adenocarcinoma (cT3N3M1c, stage IVB) and treated with osimertinib (30 mg qd) and zoledronic acid as the first-line treatment. The follow-up chest CT scans after two months showed a partial response, and brain MRI showed slightly enlarged brain metastases. Refusing gene testing by CSF and brain radiotherapy, the patient was treated with osimertinib combined with bevacizumab from February 2021. The disease progressed rapidly. Progressive disease in brain and liver lesions was observed *via* contrast CT scans performed in May 2021. A plasma sample was drawn, confirming the emergence of *EGFR* C797S with preexisting *EGFR* Ex19del by NGS, showing that the mutation allele frequency (MAF) of *EGFR* G724S had reached as high as 60.9% (**Table 2**). With consideration of resistance to osimertinib, the patient received icotinib treatment with pemetrexed. She achieved partial response in July 2021, and the NGS test revealed loss of T790M in plasma and reduction of MAF of *EGFR* G724S (AF = 10.9%). Two months later, the patient underwent another plasma NGS test that showed an elevated G724S *EGFR* mutation (AF = 41.4%) in the enlargement of liver and brain lesions. Treatment with afatinib combined with pemetrexed then commenced, and the disease progressed slowly in the following six months. In March 2022, the CT scans showed enlarged brain lesions and an increased number of liver lesions, and plasma ambulatory monitoring showed that *EGFR* G724S was manageable (AF = 17%) while co-occurring with the onset of two trans-activating mutations, T790M and C797S *EGFR*. The patient was then switched to gefitinib–osimertinib combination treatment in April 2022. CT scans revealed disease progression one month later, and NGS analysis detected a novel *EML4–ALK* fusion (**Figure 2D**). At that time, the CEA level was markedly elevated (1,272.94 ng/ml, **Figure 2E**), which indicated progressive disease. The patient died due to tumor progression in June 2022.

**Table 2:** Genetic alterations detected in plasma of Case 2.

Genes	Coding change	Alternations	MAF 2021/6/3	MAF 2021/7/22	MAF 2021/9/10	MAF 2021/11/29	MAF 2022/3/20	MAF 2022/4/15	MAF 2022/5/17
EGFR	c.2237_2255delinsT	p.E746_S752delinsV	75.30%	13.80%	53.10%	63.30%	67.40%	84.50%	72.00%
EGFR	c.2170G>A	G724S	60.90%	10.90%	41.40%	7.70%	17%	36.60%	57.10%
TP53	c.747G>T	p.R249S	27.30%	1.70%	15.60%	7.90%	17.40%	34.20%	32%
TGFBR2	c.68del	p.T23Rfs*32	17.70%	0.90%	15.00%	10.90%	17.60%	32.40%	27.00%
EGFR		amplification	CN:9.7	–	CN:4.6	CN:5.8	CN:6.6	CN:17.1	CN:9.9
EGFR	c.2390_2391delinsCT	p.C797S	3.40%	–	–	9.50%	35.00%	6.90%	5.00%
EGFR	c.2389T>A	p.c797S	2.80%	–	–	0.10%	5.10%	0.80%	0.90%
EGFR	c.2390G>C	p.C797S	0.80%	–	–	–	2.10%	–	–
EGFR	c.2152C>G	p.L718V	0.30%	–	–	–	–	–	–
LANCL2˜EGFR fusion	LANCL2:exon1˜EGFR:exon21		–	–	1.70%	–	3.30%	0.10%	0.10%
EGFR	c.2530C>G	p.L844V	–	–	0.50%	–	1.30%	0.10%	–
EGFR	c.3378G>T	p.Q1126H	–	–	0.40%	–	–	–	–
EGFR	c.2375T>A	p.L792H	–	–	–	48.40%	7.80%	27.70%	2.90%
EGFR	c.2389_2390delinsAA	p.C797N	–	–	–	–	0.80%	0.30%	1.50%
EGFR	c.2166_2170delinsATTCA	G724S	–	–	–	–	1%	13.50%	2.90%
EGFR	c.2369C>T	p.T790M	–	–	–	–	7.70%	0.90%	3.40%
APC	c.3325G>T	p.G1109C	–	–	–	–	0.50%	0.20%	–
EML4˜ALK fusion	EML4:exon6˜ALK:exon20		–	–	–	–	–	0.80%	3.20%

-, not detected; MAF, mutant allele frequency.

## Discussion

*EGFR* G724S mutation reduces osimertinib binding affinity in the context of Ex19del rather than L868R, and it has been suggested that the Ex19del/G724S coexisting mutant enhances dimerization-dependent aC helix inward conformation compared with Ex19del alone (3, 4). A case report showed that a patient with *EGFR* Ex19del/G724S mutated lung adenocarcinoma achieved disease stability with the combination of osimertinib and afatinib after osimertinib failure but then experienced disease progression within two months (5). Oztan et al. (6) presented two cases of stage IV lung adenocarcinomas harboring *EGFR* G724S concomitantly with Ex19del. One patient received carboplatin and pemetrexed, and the other was treated with nivolumab, but neither of the regimens revealed efficacy. A recent study also described a case with acquired *EGFR* G724S in the context of *EGFR* E746_S752delinsV, where PR was achieved after afatinib monotherapy with a PFS of more than 3.8 months (7). Furthermore, Wei et al. (2) reported that afatinib monotherapy induced clinical benefits in five *EGFR* Ex19del/G724S-positive patients who acquired G724S during treatment using osimertinib; the average PFS, however, was only about four months. Herein, we report two cases of *EGFR* Ex19del/G724S mutation treated with a combination of afatinib and chemotherapy. Primary lesions detected concomitant G724S and Ex19del mutation in Case 1, where significant reduction of the primary and metastatic lesions was found, and PR with a PFS of more than 16 months was achieved. The *EGFR* G724S in Case 2 was found to be a potential driver of acquired resistance to osimertinib. Treated by afatinib combined with chemotherapy, disease control was achieved for six months. Compared with afatinib monotherapy, patients harboring Ex19del/G724S mutation respond better to the combination therapy of afatinib and chemotherapy, providing a treatment strategy for this subset of patients. Moreover, the combination of afatinib with chemotherapy did not increase the incidence and severity of adverse events.

An analysis of the acquired mutation profile in patients treated using afatinib revealed the mechanism of resistance in Ex19del/G724S-positive patients who had available rebiopsy samples. In Case 1, a female patient with stage IV recurrent lung adenocarcinoma, who had baseline *EGFR* Ex19del concomitant with G724S, received afatinib as first-line treatment; there was no drug resistance gene found until she died. In contrast, in Case 2, a female patient with baseline *EGFR* Ex19delins progressed after five months of osimertinib treatment. *EGFR* C797S and G724S were subsequently identified as osimertinib resistance mutations. After six months of afatinib treatment, *EML4*–*ALK* fusion emerged, which led to the resistance to afatinib (**Table 2**). Wei et al. (2) reported that one patient acquired *MET* amplification in addition to *EGFR* T790M at treatment with afatinib. Our case report contributes to our understanding of afatinib-acquired resistance mechanisms in Ex19del/G724S-positive patients.

Furthermore, the *TP53* mutation is the most common concomitant gene in *EGFR*-mutated NSCLC. The meta-analysis revealed an association between concurrent *TP53* mutations and worse outcomes in advanced NSCLC treated with EGFR-TKIs (8). Mutations in exon 4 or 7 of *TP53* served as independent negative prognostic factors for progression-free and overall survival in NSCLC patients (9). The short clinical benefit time of Case 2 may have been caused by the concomitant presence of exon 7 of *TP53* R249S, and that of Case 1 with exon 5 of *TP53* R158del. Currently, how to determine the optimal treatment strategies for patients with concomitant TP53 mutation in *EGFR*-mutant NSCLC is under investigation. Here, we innovatively combined EGFR-TKI with chemotherapy to improve the prognosis of *TP53* concurrent mutation patients, yet patients with uncommon *EGFR* gene mutations remain to be further explored. In the current cases, CEA is not a specific marker of malignant tumors; it only has auxiliary value in diagnosis, and the comprehensive molecular profiling of the tumors could assist in precise treatment beyond standard tests.

## Conclusions

In conclusion, we described for the first time the effectiveness and safety of afatinib in combination with chemotherapy in the cases of *EGFR* Ex19del/G724S-positive patients. Treatment efficacy in the two patients may provide a new horizon for this *EGFR* rare mutation. Plasma cfDNA analysis reveals that *ALK* fusion is one cause of afatinib resistance in G724S-positive patients. In this possible future scenario, seriate cfDNA analysis could be a useful tool for guiding treatment selection to manage different disease clones.

## Data availability statement

The datasets presented in this study can be found in online repositories. The names of the repository/repositories and accession number(s) can be found in the article/supplementary material.

## Ethics statement

The studies involving human participants were reviewed and approved by the Ethical Committee of Guangxi Medical University Cancer Hospital. The patients/participants provided their written informed consent to participate in this study.

## Author contributions

HW and QY were involved in the diagnostic flow and patient follow-up, LS wrote this article, and the manuscript was edited by CL. Collection and assembly of data: QH, LD, YZ, NR, and XH. All authors contributed to the article and approved the submitted version.

## Funding

This work was supported by the Basic Competence Promotion Project for Young and Middle-aged Teachers in Guangxi, China (CN) (no. 2019KY0137).

## Acknowledgments

We thank Congcong Li of Nanjing Geneseeq Technology Inc., Nanjing, Jiangsu, China, for his assistance with sample information collection.

## Conflict of interest

Author QH was employed by Nanjing Geneseeq Technology Inc.

The remaining authors declare that the research was conducted in the absence of any commercial or financial relationships that could be construed as a potential conflict of interest.

## Publisher’s note

All claims expressed in this article are solely those of the authors and do not necessarily represent those of their affiliated organizations, or those of the publisher, the editors and the reviewers. Any product that may be evaluated in this article, or claim that may be made by its manufacturer, is not guaranteed or endorsed by the publisher.
